# Integrative methylation score to identify epigenetic modifications associated with lipid changes resulting from fenofibrate treatment in families

**DOI:** 10.1186/s12919-018-0125-x

**Published:** 2018-09-17

**Authors:** Biqi Wang, Anita L. DeStefano, Honghuang Lin

**Affiliations:** 1 Department of Biostatistics, Boston University School of Public Health, 801 Massachusetts Avenue, Boston, MA 02118 USA; 2 National Heart, Lung, and Blood Institute’s and Boston University’s Framingham Heart Study, 73 Mount Wayte Avenue, Framingham, MA 01702 USA; 3 Section of Computational Biomedicine, Department of Medicine, Boston University School of Medicine, 72 E Concord St, B-616, Boston, MA 02118 USA

## Abstract

Epigenome-wide association studies (EWAS) have traditionally focused on the association test of single epigenetic markers with complex traits. However, it is possible that multiple cytosine-phosphate-guanine (CpG) sites at the same locus could jointly exert their effects on human traits. Therefore, a region-based test that combines multiple markers could be more powerful. We used 2 different region-based tests to investigate the association between changes in DNA methylation and drug response, including the median methylation level test (MMLT) and sequence kernel association test (SKAT). No genes were found to be significantly associated with the drug response (for triglycerides, the false discovery rate ranged from 0.855 to 0.999; for high-density lipoprotein cholesterol, and the false discovery rate ranged from 0.584 to 0.915). Further evidence is needed to explore potential application of gene-level methylation association analysis.

**Electronic supplementary material:**

The online version of this article (10.1186/s12919-018-0125-x) contains supplementary material, which is available to authorized users.

## Background

Considerable interindividual variabilities of responses have been observed in people who take lipid-lowering drugs, underscoring the importance of genetic variants to the drug response. For instance, common genetic polymorphisms in the apolipoprotein B (*APOB*) gene are associated with the capability of transferring triglyceride and cholesteryl esters during lipoprotein metabolism [1]. Moreover, DNA methylation could be altered by lipid-lowering drugs, which can interrupt methionine biosynthesis through multiple pathways [2]. It was found that the methylation level on *CPT1A* was significantly associated with triglyceride and high-density lipoprotein cholesterol (HDL-C) in European populations [3].

Das et al. performed an epigenome-wide association study (EWAS) to test the association of each individual cytosine-phosphate-guanine (CpG) site with lipid levels before and after 3 weeks of fenofibrate treatment [2]. No significant association was found. It is possible that multiple CpG sites on the same locus, each one with small effect, could jointly exert their effects, which could not be identified by the single-CpG site analysis [4]. It is thus interesting to explore region-based tests, which could use the information from multiple CpG sites and examine their joint effects on drug response. Such analysis can be a complement to current single-CpG association studies. The objective of this study is to investigate whether region-based changes of methylation profiles are associated with lipid changes induced by fenofibrate treatment in a family-based population.

## Methods

### Data processing

The family data from the Genetics of Lipid Lowering Drugs and Diet Network (GOLDN) study was obtained via GAW20. A detailed description of the GOLDN study design can be found elsewhere [3]. Briefly, the study is a family-based, open-label, and one arm clinical trial assessing the lipid-lowering-drug effect on human genomics after a treatment of fenofibrate (160 mg) for 3 weeks. Participants included in current analyses met these3 criteria: (a) having qualified methylation data both before and after the 3 weeks of treatment; (b) having 1 or 2 lipid measurements at both pre- and posttreatment times; (c) having all the covariate data (such as age, sex, field center, and smoking status) at entry of the trial. There were 446 participants of European ancestry from 139 families that met these criteria.

DNA methylation was quantified by the Illumina Infinium HumanMethylation450K BeadChip using CD4+ T cells both pre- and posttreatment. The methylation level of each CpG site was estimated as a continuous variable ranging from 0 (not methylated) to 1 (fully methylated). Quality control of the methylation data was conducted by samples and probes separately. A multidimensional scaling plot was used to identify outliers among samples; no samples were excluded. For quality control of probes, the following filters were applied: exclusion of probes with single-nucleotide polymorphisms (SNPs) with minor allele frequency > 0.01 under the actual CpG sites or at the single base extension (17,297 probes), or cross-reacted to multiple targets (29,233 probes) [5]. After quality control, 446 samples with 423,180 probes remained. Beta-mixture quantile normalization was conducted to control biases from 2 different types of probes of the 450 K methylation array [6]. In addition, the first 20 principal components were calculated separately in pre- and posttreatment methylation data to control for potential confounders.

Lipid profiles were measured from blood after an overnight fast both before and after 3 weeks of intervention. Details about lipid measurement have been described in the previous GOLDN publication [2]. Gender, field center (Minnesota or Utah), and metabolic syndrome (yes or no) were binary variables. Metabolic syndrome was defined according to National Cholesterol Education Program (NCEP) guidelines from American Heart Association (AHA)/National Heart, Lung, and Blood Institute (NHLBI) 2004 meeting. Smoking status was categorized by never, past, and current smokers. Age and lipid were continuous variables in our analysis.

### Median methylation level test of genes

To better capture the effect of gene-level methylation, we defined a gene region by including all the CpG sites between 100 kb of the first transcript start site (TSS), and 100 kb downstream of the last TSS of the gene [7]. The median methylation level of all the CpG sites within a region was calculated as a representation of the overall methylation pattern of the gene [7]. A total of 21,231 unique gene regions were identified, which encompassed more than 300,000 CpG sites. The TSSs were retrieved from GENCODE release 19, which was downloaded from the UCSC Genome Browser. Among the 21,231 unique genes, 894 were not among the UCSC known genes, and an additional 4602 genes did not contain any qualified CpG sites. Thus only median methylation levels for 15,735 genes were calculated for each sample. Both the pre- and posttreatment methylation matrices were composed of 446 samples.

The change of median gene region methylation was calculated as pretreatment median methylation minus posttreatment median methylation. A linear mixed-effect regression model was used to investigate the association between change in methylation median and natural logarithm transformed change of either triglyceride or HDL-Cafter 3 weeks of fenofibrate intervention. The outcome variable was natural log-transformed lipid change, while the change in median methylation was treated as an independent variable. Age, gender, field center, metabolic syndrome, smoking status, and pre– or post–principal components that were significantly associated with changes of lipid were treated as fixed effects, and the family structure was treated as a random effect. The analysis was conducted using the *lmekin* function in the R *comex* package, and the significance threshold was defined as Benjamin-Hochberg false discovery rate q value equal to 0.05 [8].

### Sequence kernel association test of genes

The sequence kernel association test (SKAT) method is one of the variance-component approaches that tests for associations by evaluating the distribution of genetic effects for a group of variants. It can be used to assess the association of a continuous or binary trait with a set of CpG sites in a similar fashion to a gene-level analysis in genome-wide genotyping or sequencing data [9]. For example, given a continuous trait such as the natural log-transformed change in triglyceride or HDL-C, the change of methylation level at single CpG sites would have a score statistic that was calculated by the marginal linear mixed-effect model adjusting for covariates and family structure. Multiple CpG sites could then be combined as a single CpG set. Considering such a set, SKAT for methylation sites uses a joint statistic that is an unweighted (all weights were equal to 1) sum of squares of the single CpG sites score statistics for the set. The joint statistic asymptotically follows a mixture chi-square distribution and its *p* value can be computed analytically quickly. Here we used the same 15,735 gene regions as above to identify differentially methylated genes. The analysis was conducted using R package *seqMeta* and the significance level was defined as the same as in the median methylation analysis [10].

Sensitivity analysis was performed to address whether results would change when we applied more weights to the CpG sites that changed as a consequence of drug response. For example, the changes of methylation were treated as a continuous variable ranging from − 1 to 1 (where the closer to 0, the less likely the methylation changed). We also computed the “methylation changing dosage,” which was defined as methylation changes plus 1, which ranged from 0 to 2. Thus a CpG site with most dosage around 1 meant that the methylation level remained the same (and methylation dosage frequency would close to 0.5), whereas a CpG with most dosages at 0 (or 2) (the methylation dosage frequency would be small, or even rare) meant that the methylation level actually changed between pre- and posttreatment. So, the default setting in *seqMeta* (which gave the rare alleles more weights) will add more weight to CpG sites with significant change of methylation levels, and less weight to CpG sites with little or change of methylation levels.

## Results

Table 1 summarizes the characteristics of the 446 participants (mean age: 47.3 years). The number of women was slightly larger than the number of men. The triglyceride level tended to decrease while HDL-C increased after 3 weeks of fenofibrate intervention.

**Table 1:** Characteristics of 446 participants included in this analysis

Characteristics	N (%) or mean ± SD
Number of participants	446
Age (years)	47 ± 16
Women	230 (51.6)
Field centers	
Minnesota	201 (45.1)
Utah	245 (54.9)
Metabolic syndrome^a^	179 (40.1)
Smoking status	
Never smoker	316 (70.9)
Past smoker	100 (22.4)
Current smoker	30 (6.7)
Changes of triglycerides (mg/dL)	48.9 ± 54.6
Changes of high-density lipoprotein cholesterol (mg/dL)	−2.9 ± 5.1

^a^Metabolic syndrome was defined according to NCEP guidelines from AHA/NHLBI 2004 meeting

Tables 2 and 3 show the top 10 genes associated with the natural logarithm changes of triglyceride and HDL-C, respectively. We did not find any genes significantly associated with triglyceride or HDL-C after correction for multiple testing. Interestingly, *CPT1A* was among one of the top triglyceride genes by SKAT, although it did not reach the predefined genome-wide significance.

**Table 2:** Top 10 genes associated with natural logarithm change in triglycerides

MMLT						SKAT^a^				
Genes	*N*	Effect size	SE	*p* Value	FDR q value	Genes	*N*	Q-statistic	*p* Value	FDR q value
*CSHL1*	3	1.29	0.35	2.02E-04	0.999	*RPL29*	1	117.39	1.59E-03	0.855
*C22orf39*	4	−1.89	0.56	7.74E-04	0.999	*GJB5*	2	104.51	2.95E-03	0.855
*C14orf166*	4	2.63	0.81	1.19E-03	0.999	*TBX20*	2	104.51	2.95E-03	0.855
*RPL29*	1	0.91	0.29	1.41E-03	0.999	*WDR92*	7	436.86	3.75E-03	0.855
*PRPH*	42	0.95	0.30	1.57E-03	0.999	*CPT1A*	6	439.50	3.92E-03	0.855
*GSG1*	2	−1.28	0.41	2.07E-03	0.999	*MIR1266*	2	51.56	5.29E-03	0.855
*LPIN2*	41	−0.95	0.32	2.55E-03	0.999	*TIMP4*	5	191.94	7.11E-03	0.855
*IP6K3*	124	−1.26	0.42	2.61E-03	0.999	*ZNF788*	5	191.94	7.11E-03	0.855
*PPIC*	53	−0.90	0.31	3.63E-03	0.999	*GSG1*	2	105.07	8.14E-03	0.855
*RHBDL3*	21	0.49	0.17	3.94E-03	0.999	*CRYGA*	3	115.23	1.06E-02	0.855

FDR, false discovery rate; N, number of CpG sites in the gene set

^a^The weight of SKAT all equaled 1

**Table 3:** Top 10 genes associated with natural logarithm change in HDL-C

MMLT						SKAT^a^				
Genes	N	Effect size	SE	*p* Value	FDR q value	Genes	N	Q-statistic	*p* Value	FDR q value
*ZKSCAN8*	4	0.62	0.16	7.42E-05	0.584	*KRT222*	6	1271.43	5.97E-03	0.915
*ZSCAN16*	4	0.62	0.16	7.42E-05	0.584	*FAM211A*	5	840.88	1.00E-02	0.915
*MAGI2*	67	0.27	0.08	8.12E-04	0.999	*ZKSCAN8*	4	1095.82	1.13E-02	0.915
*CAPN3*	30	0.29	0.09	8.72E-04	0.999	*ZSCAN16*	4	1095.82	1.13E-02	0.915
*AK5*	37	0.27	0.08	8.78E-04	0.999	*HEATR5B*	1	552.38	1.28E-02	0.915
*KCTD14*	37	0.27	0.08	8.78E-04	0.999	*IFT27*	1	552.38	1.28E-02	0.915
*PTP4A2*	91	0.29	0.09	1.44E-03	0.999	*ZNF567*	1	552.38	1.28E-02	0.915
*GSTTP1*	28	−0.45	0.14	1.45E-03	0.999	*ZNF337*	4	983.95	1.28E-02	0.915
*FOXI1*	27	0.31	0.10	1.64E-03	0.999	*CD300E*	3	1014.54	1.40E-02	0.915
*VPS37A*	4	0.88	0.28	1.76E-03	0.999	*SORCS1*	9	1010.61	1.60E-02	0.915

FDR, false discovery rate; N, number of CpG sites in the gene set

^a^The weight of SKAT all equaled 1

**Fig. 1:**
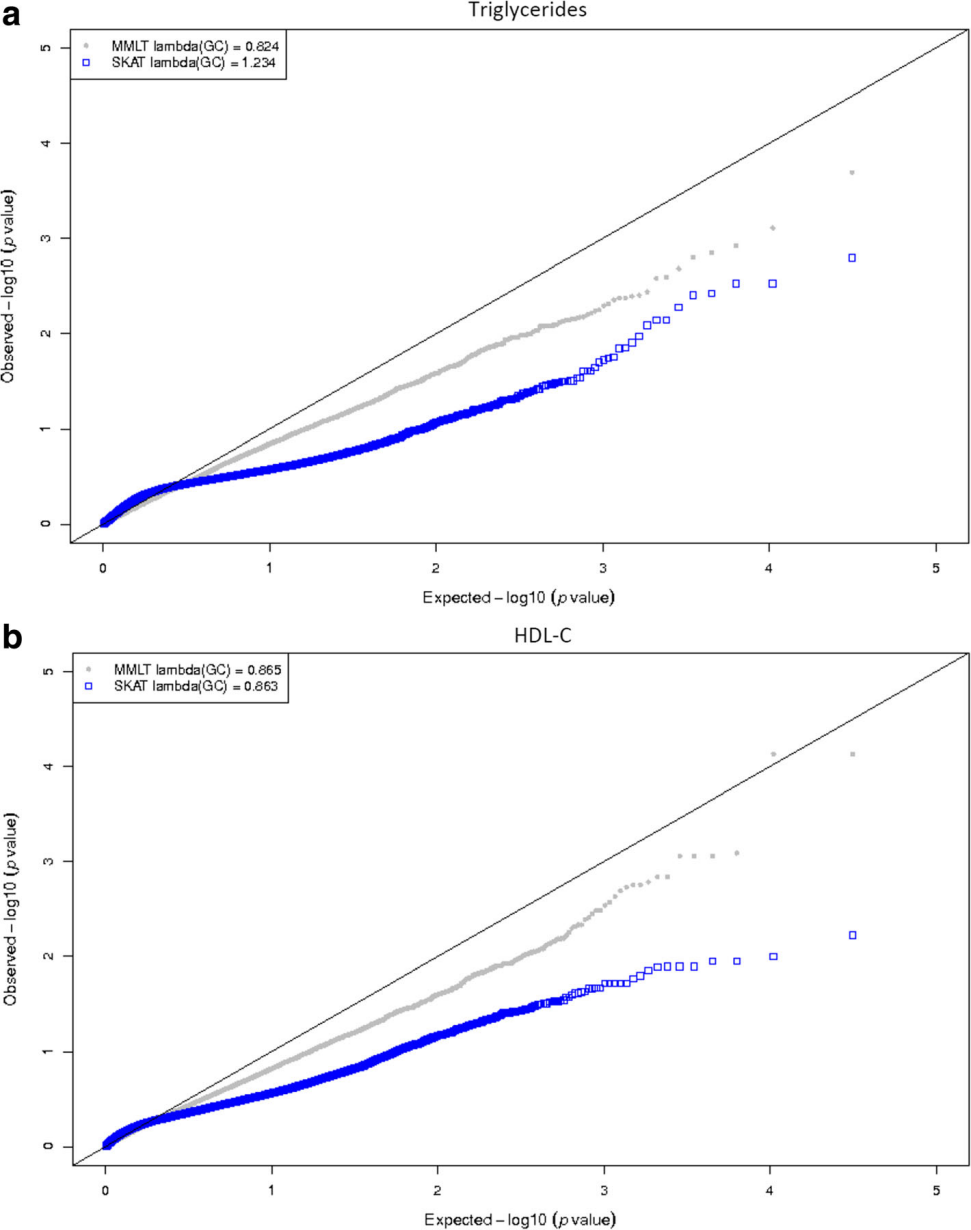
Quantile–quantile plots of association tests by MMLT and SKAT for natural logarithm changes of triglyceride or HDL-C. Shown in panel **a** is the quantile-quantile plot for triglycride, in panel **b** is the plot for HDL-C

**Fig. 2:**
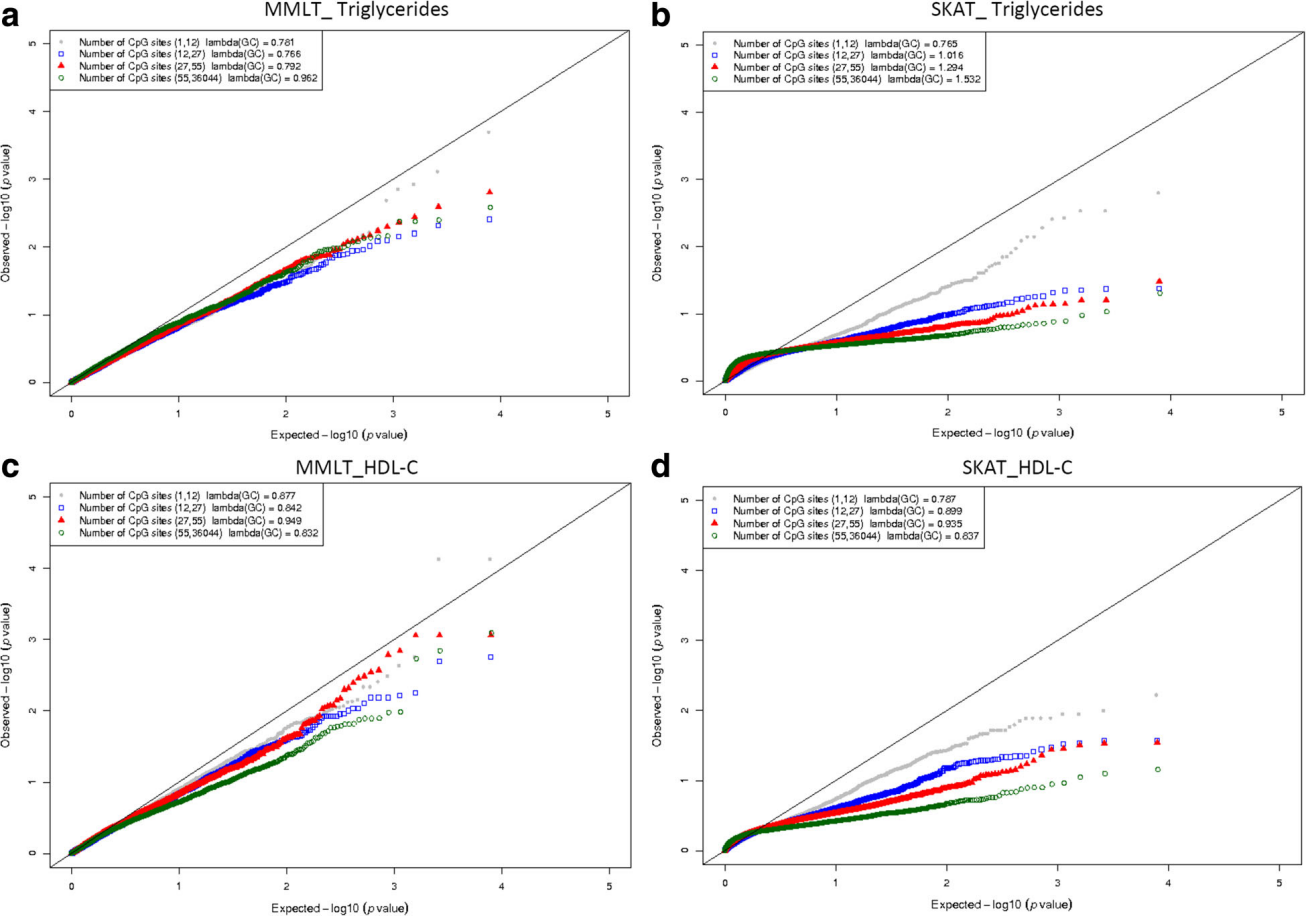
Quantile–quantile plots by numbers of CpG sites in MMLT and SKAT for natural logarithm changes of triglyceride or HDL-C. Shown in panel **a** is the quantile-quantile plot for triglyceride by MMLT, in panel **b** is for triglycerides by SKAT, in panel **c** is for HDL-C by MMLT, and in panel **d** is for HDL-C by SKAT

Quantile-quantile plots were generated to identify whether the Type I error was inflated or deflated by median methylation level test (MMLT) and SKAT (Fig. 1). The plot of SKAT in natural logarithm changes in triglyceride show some inflation with a genomic control factor of 1.234, while others were deflated. Further plotted by the numbers of CpG sites in the gene region (which was categorized by the quartiles of CpG sites) showed that for SKAT in natural logarithm changes in triglyceride, the plot seemed to be more inflated in the gene regions with more CpG sites (Fig. 2).

Sensitivity analysis showed that no genes were significantly associated with natural logarithm changes of triglycerides or HDL-C (see Additional file 1*:* Table S1 and Table S2), but with relatively lower Type I error than the unweighted SKAT method (see Additional file 1*:* Figures S1 and S2).

## Discussion

In the present study, we used 2 region-based association tests to examine epigenetic modifications associated with changes in lipid levels induced by fenofibrate in a family-based population. No genes were significantly associated with the change in triglyceride or HDL-C after 3 weeks of intervention, similar to the single CpG test.

Two region-based methods were evaluated in our study. The Type I error seemed deflated in testing by MMLT across lipids changes. When the number of CpG sites increased, the Type I error slightly increased but still remained deflated. Our results suggest that MMLT needs a relatively large number of changing CpG sites to capture the overall methylation profile of gene. The method has less power if only a small number of CpG sites changed as it only calculated the median level. In contrast, the Type I error inflated along with the number of CpG sites in the gene when tested by SKAT, suggesting that noises might be introduced by including too many irrelevant CpG sites. Moreover, the Type I error decreased after assigning more weight to the changed CpG sites, which also indicated unchanged CpG sites might create “noise” and dilute the effect of changed CpG sites.

Although neither method identified gene-level significant methylation profiles associated with lipid changes, SKAT identified *CPT1A*as a potentially interesting gene associated with the changes of triglyceride. *CPT1A* is a gene responded to a key enzyme in the carnitine-dependent transport across the mitochondrial inner membrane and its deficiency results in a decreased rate of fatty acid beta-oxidation. The MMLT method has lower power compared to SKAT when the directions of effects are different [11]. For example, a gene could contain some CpG sites that were positively associated with the drug response, and some CpG sites that were negatively associated with the drug response. The overall signal could be attenuated because the 2 groups of CpG sites have opposite effects. In contrast, SKAT is particularly powerful for identifying significant gene sets in such a case, because it takes into account of the direction of effects in the model [12].

We acknowledge several limitations of our study. Given the limited access to the GOLDN study, we might have missed some important factors related to batch effects or potential confounders. In addition, 3 weeks of intervention might not be long enough to observe significant change in DNA methylation. Additional observations are needed to observe methylation trajectory [13].

## Conclusions

We found limited evidence for region-based analysis in identifying methylation genes associated with drug response. Longer and multipoint observations could provide additional insights into the effects of pharmaceutical interventions.

## Additional file

Additional file 1: **Table S1.** Top 10 genes associated with change in triglycerides. **Table S2.** Top 10 genes associated with change in HDLc. **Figure S1.** Quantile-quantile plots of association tests by MMLT and weighted SKAT for natural logarithm changes of triglyceride or HDLc. The weight of SKAT was the algorithm of minor allele frequency. **Figure S2.** Quantile-quantile plots by numbers of CpGs in MMLT and weighted SKAT for for natural logarithm changes of triglyceride or HDLc. (PDF 600 kb)
