# Supplementary material for: Integrative methylation score to identify epigenetic modifications associated with lipid changes resulting from fenofibrate treatment in families
Source: BMC Proc. 2018 Sep 17;12(Suppl 9):28. doi: 10.1186/s12919-018-0125-x (PMC6157127; doi:10.1186/s12919-018-0125-x)
Supplement: Supplementary file 1 — Table S1. Top 10 genes associated with change in triglycerides. Table S2. Top 10 genes associated with change in HDLc. Figure S1. Quantile-quantile plots of association tests by MMLT and weighted SKAT for natural logarithm changes of triglyceride or HDLc. The weight of SKAT was the algorithm of minor allele frequency. Figure S2. Quantile-quantile plots by numbers of CpGs in MMLT and weighted SKAT for for natural logarithm changes of triglyceride or HDLc. (PDF 600 kb) [file 12919_2018_125_MOESM1_ESM.pdf]

Additional file for Integrative methylation score to identify epigenetic modifications associated with lipid changes due to fenofibrate treatment in families

Biqi Wang[1], Anita DeStefano[1], Honghuang Lin[2, 3, §]

Affiliations:

1 Department of Biostatistics, Boston University, Boston, MA, USA
2 National Heart, Lung, and Blood Institute's and Boston University's Framingham Heart Study, Framingham, MA
3 Section of Computational Biomedicine, Department of Medicine, Boston University School of Medicine, Boston, MA

Emails:

BW: wangbiqi@bu.edu

AD: adestef@bu.edu

HL: hhlin@bu.edu

[§] Corresponding author

Table S1. Top 10 genes associated with change in triglycerides

		Median methylation level test (MMLT)				Sequence kernel association test (SKAT)				
**Genes**	**N**	**Effect size**	**S.E.**	**p value**	**FDR q value**	**Genes**	**N**	**Q-statistic**	**p value**	**FDR q value**
CSHL1	3	1.29	0.35	2.02E-04	0.999	***RPL29***	1	6.07E-10	1.59E-03	0.942
C22orf39	4	-1.89	0.56	7.74E-04	0.999	***WDR92***	7	2.10E-09	3.78E-03	0.942
C14orf166	4	2.63	0.81	1.19E-03	0.999	***GJB5***	2	4.65E-10	4.32E-03	0.942
RPL29	1	0.91	0.29	1.41E-03	0.999	***TBX20***	2	4.65E-10	4.32E-03	0.942
PRPH	42	0.95	0.30	1.57E-03	0.999	***MIR1266***	2	1.42E-10	5.28E-03	0.942
GSG1	2	-1.28	0.41	2.07E-03	0.999	***CPT1A***	6	2.13E-09	5.39E-03	0.942
LPIN2	41	-0.95	0.32	2.55E-03	0.999	***CRYGA***	3	7.55E-10	6.38E-03	0.942
IP6K3	124	-1.26	0.42	2.61E-03	0.999	***SLC5A9***	3	5.65E-10	8.94E-03	0.942
PPIC	53	-0.90	0.31	3.63E-03	0.999	***GSG1***	2	7.26E-10	1.01E-02	0.942
RHBDL3	21	0.49	0.17	3.94E-03	0.999	***TIMP4***	5	5.53E-10	1.01E-02	0.942

The weight of SKAT was the algorithm of minor allele frequency.

Table S2. Top 10 genes associated with change in HDLc

Median methylation level test (MMLT)						Sequence kernel association test (SKAT)				
**Genes**	**N**	**Effect size**	**S.E.**	**p value**	**FDR q value**	**Genes**	**N**	**Q-statistic**	**p value**	**FDR q value**
ZKSCAN8	4	0.62	0.16	7.42E-05	0.584	***VPS25***	30	2.43E-07	6.58E-03	0.916
ZSCAN16	4	0.62	0.16	7.42E-05	0.584	***ZFP69B***	33	2.45E-07	7.03E-03	0.916
MAGI2	67	0.27	0.08	8.12E-04	0.999	***SYNE1***	64	1.67E-07	7.89E-03	0.916
CAPN3	30	0.29	0.09	8.72E-04	0.999	***ACHE***	18	1.25E-08	8.80E-03	0.916
AK5	37	0.27	0.08	8.78E-04	0.999	***KRT222***	6	5.76E-09	9.38E-03	0.916
KCTD14	37	0.27	0.08	8.78E-04	0.999	***CD300E***	3	3.73E-09	9.51E-03	0.916
PTP4A2	91	0.29	0.09	1.44E-03	0.999	***UCN3***	49	1.78E-07	1.05E-02	0.916
GSTTP1	28	-0.45	0.14	1.45E-03	0.999	***CRBN***	87	1.43E-06	1.08E-02	0.916
FOXI1	27	0.31	0.10	1.64E-03	0.999	***NCLN***	87	1.43E-06	1.08E-02	0.916
VPS37A	4	0.88	0.28	1.76E-03	0.999	***ZNF200***	77	1.43E-06	1.09E-02	0.916

The weight of SKAT was the algorithm of minor allele frequency.

A. Triglycerides | B. HDLc

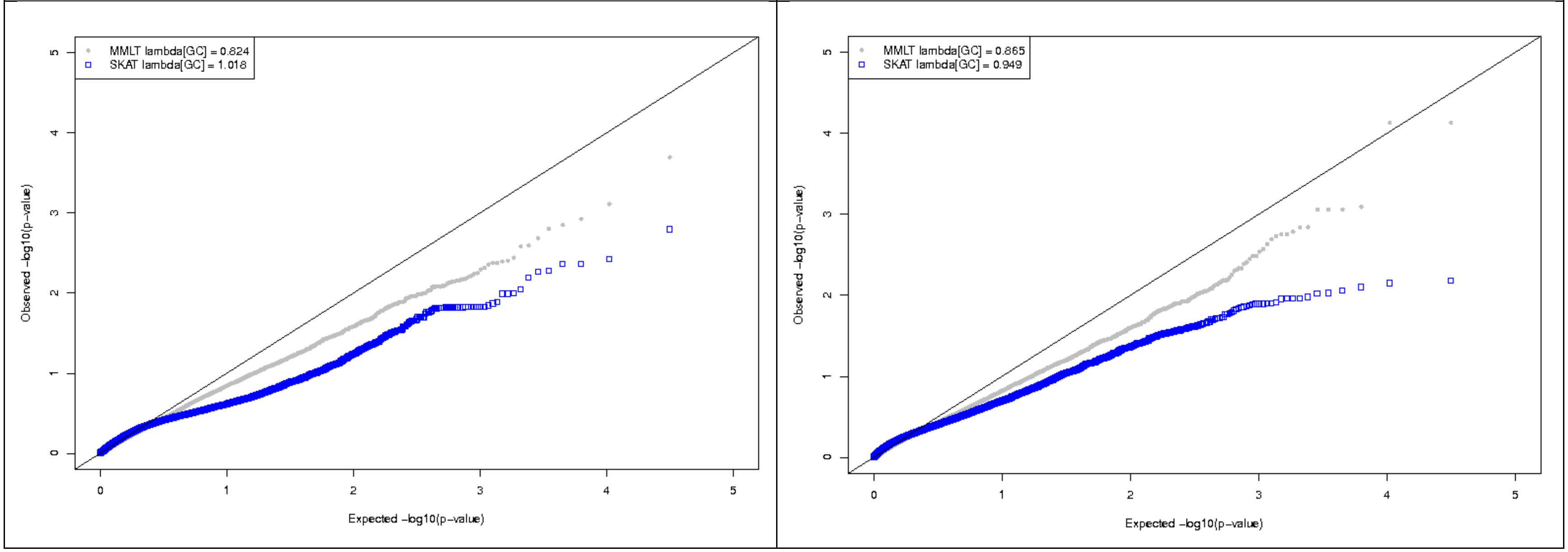

Figure S1. Quantile-quantile plots of association tests by MMLT and weighted SKAT for natural logarithm changes of triglyceride or HDLc. The weight of SKAT was the algorithm of minor allele frequency.

A. MMLT_ Triglycerides

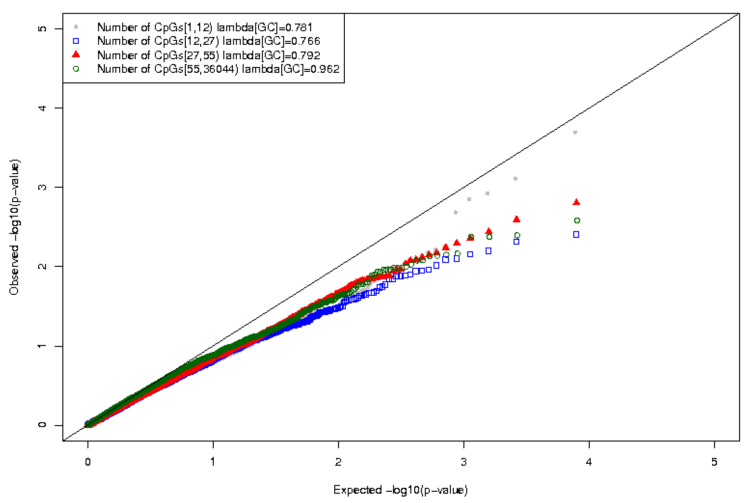

B. SKAT_ Triglycerides

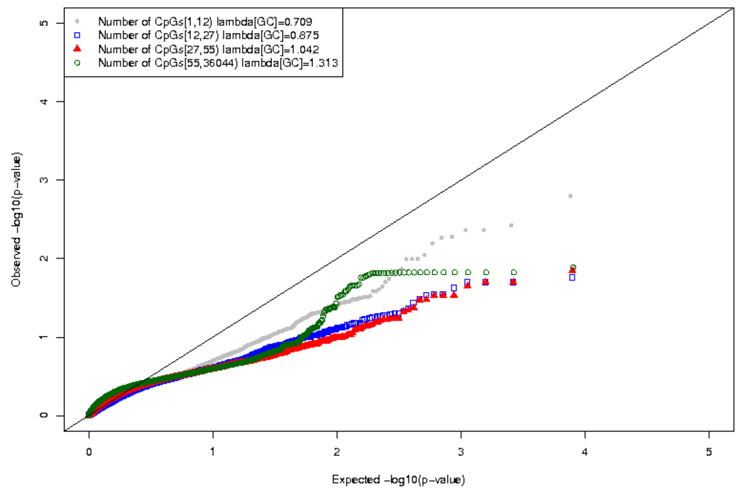

C. MMLT_HDLc

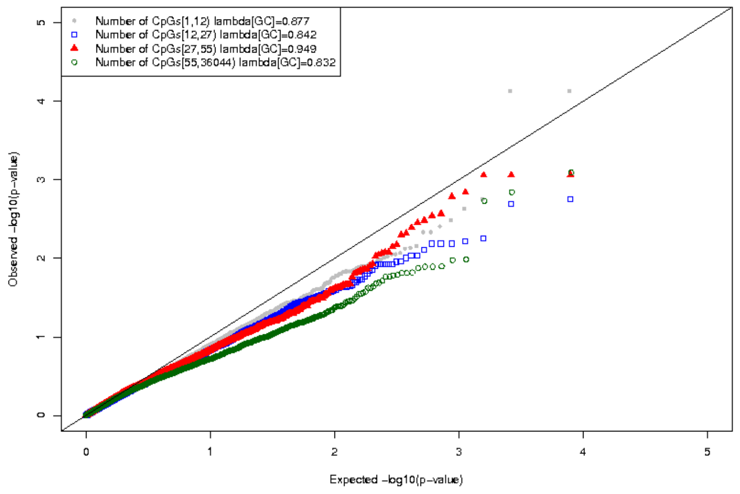

D. SKAT_HDLc

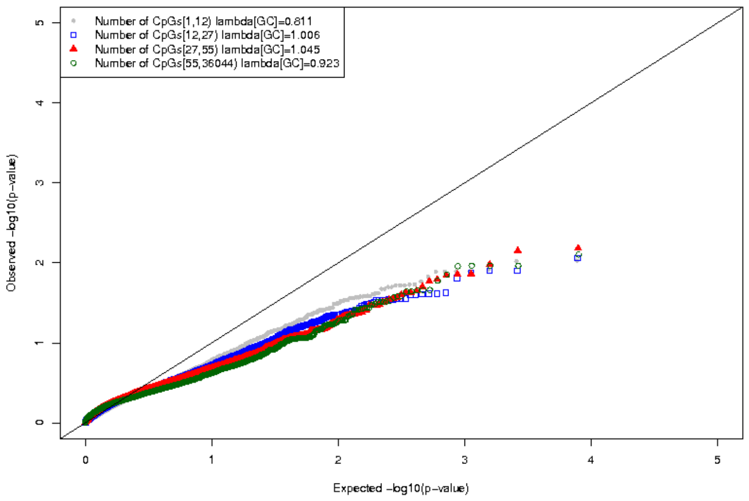

Figure S2. Quantile-quantile plots by numbers of CpGs in MMLT and weighted SKAT for for natural logarithm changes of triglyceride or HDLc.
